# Le dermatofibrosarcome de Darier et Ferrand, une tumeur cutanée particulière: à propos de 32 cas et revue de la littérature

**DOI:** 10.11604/pamj.2014.19.196.4470

**Published:** 2014-10-24

**Authors:** Driss Elamrani, Hatim Droussi, Samira Boukind, Keltoum Elatiqi, Meriem Dlimi, Yassine Benchamkha, Saloua Ettalbi

**Affiliations:** 1 Service de Chirurgie Plastique et Brûlés, CHU Mohammed VI, Marrakech, Maroc; 2 Laboratoire d'Anatomie, Faculté de Médecine et de Pharmacie, Université Cadi Ayyad, Marrakech, Maroc

**Keywords:** Chirurgie reconstructrice, dermatofibrosarcome de Darier et ferrand, sarcome, tumeurs cutanées, reconstructive surgery, dermatofibrosarcoma protuberans, sarcoma, skin tumors

## Abstract

Le dermatofibrosarcome (DFS) est une tumeur fibreuse de la peau, de croissance lente, à très haut risque de récidive locale, mais à potentiel métastatique faible. A partir d'une étude rétrospective étalée sur une période de 5 ans (décembre 2008 - décembre 2013), nous avons analysé les caractéristiques épidémiologiques et cliniques, le délai de diagnostic, le type de thérapeutique et le devenir de 32 patients présentant des tumeurs de Darier et Ferrand histologiquement prouvées. Parmi les 32 patients, 10 se sont présentés initialement au service pour une récidive tumorale. Une discrète prédominance masculine a été notée. Les DFS touchent préférentiellement l'adulte jeune. Le délai diagnostique observé est en moyenne de 4 ans. Le tronc est la localisation préférentielle (60%), suivi par les extrémités proximales (30%). Les 32 patients ont été traités par exérèse chirurgicale avec une marge de 5cm en surface, emportant en profondeur une barrière anatomique saine. La couverture de la perte de substance (PDS) a été réalisée après confirmation anatomopathologique du caractère carcinologique de l'exérèse, et a fait appel à différents moyens allant de la greffe cutanée aux lambeaux musculo –cutanés libres. L’évolution a été marquée par la survenue d'une récidive tumorale chez 8 patients (3 cas parmi les tumeurs vues en première intention et 5 cas parmi les tumeurs vues en récidive) et les résultats ont été jugés satisfaisants sur le plan esthétique et fonctionnel. Le DFS de Darier et Ferrand est une tumeur dont le pronostic et le risque évolutif sont principalement liés au délai diagnostic et la qualité de la première exérèse. Le diagnostic tardif, rend difficile la chirurgie d'exérèse et de reconstruction Les possibilités de guérison en cas de chirurgie primaire bien conduite sont significativement supérieures à celles d'une chirurgie de rattrapage. L'amélioration du pronostic passe par une prise en charge multidisciplinaire précoce et codifiée d'où l'intérêt de la sensibilisation et de l'information du médecin généraliste pour le diagnostic précoce et l'orientation correcte de ces malades vers des centres spécialisés.

## Introduction

Le dermatofibrosarcome de Darier et Ferrand (DFS) est une tumeur cutanée mésenchymateuse à développement intradermique décrite pour la première fois par Taylor [1] comme une tumeur sarcomateuse. C'est une tumeur, située entre le pôle de bénignité du très fréquent et inoffensif fibrome cutané et le pôle de malignité du fibrosarcome cutané vrai. Sa transformation sarcomateuse franchement maligne avec métastase est exceptionnelle. Cette tumeur, dont la fréquence est non négligeable dans les pays africains [2], pose encore plusieurs problèmes en rapport avec: Son caractère méconnu par la grande majorité des médecins généralistes et même certains spécialistes, son aspect clinique trompeur évoquant surtout une cicatrice chéloïde (fréquente chez la population africaine) et responsable souvent d'un retard diagnostic, sa gravité liée à son agressivité locale et son potentiel destructeur, son caractère récidivant si le traitement initial n'a pas respecté les règles rigoureuses qu'exige toute prise en charge de cette tumeur particulière. A fin de répondre à tous ces problèmes, ce travail propose une analyse rétrospective d'une série de 32 cas où les auteurs partagent leur expérience rapportant les différentes caractéristiques épidémiologiques, cliniques, pronostiques et thérapeutique de cette tumeur cutanée si particulière.

## Méthodes

Notre étude est rétrospective étalée sur 5 ans de Décembre 2008 à Décembre 2013. Cette étude inclue tout patient consultant pour DFS confirmés histologiquement, jamais traités ou vus en récidive après un traitement initial réalisé ailleurs. Le nombre de cas colligés est de 32 cas, dont 10 tumeurs récidivées. La collecte des données à partir des dossiers a été réalisée sur une fiche d'exploitation préalablement préparée. Pour chaque dossier, les éléments suivants ont été pris en compte: l’état civil, le siège de la tumeur, la taille de la tumeur, le délai de survenue, le nombre de récidives, le délai de récidive, les signes cliniques, le protocole opératoire, les résultats de l'examen anatomopathologique et les complications post-opératoires.

## Résultats

**Figure 1:**
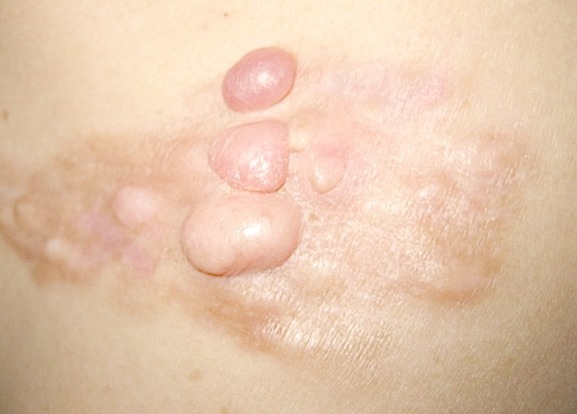
Plaque parsemée de nodules, blanc-jaunâtre de la région scapulaire, évocatrice de dermatofibrosarcome de Darrier et Ferand

Une légère prédominance masculine a été retrouvée: 18 hommes et 12 femmes. Au moment où le diagnostic a été posé, les patients étaient âgés de 23 à 71 ans avec une moyenne de 39,2 ans. Dans notre série, nous n'avons pas retrouvé d'antécédents familiaux, ni néoplasiques, ni facteurs déclenchants associés. Le délai diagnostique correspondant à la période écoulée entre l'apparition de la lésion et le diagnostic de DFS est en moyenne de 4,8 ans pour les 24 patients qui ont pu le préciser (Tableau 1) Les localisations tumorales dans notre étude, intéressent préférentiellement le tronc et les extrémités proximales des membres (Tableau 2). La lésion initiale est une petite plaque fibreuse rosée ou un nodule ferme enchâssé dans la peau, qui s’étend lentement pour aboutir à une formation tumorale indolore évocatrice: brun ou rose, et parfois télengiectasique (Figure 1). Les nodules peuvent s'exulcérer. La lésion unique se présente comme un placard dermohypodermique, indolore spontanément et à la palpation, à surface bosselée mobile sur les plans profonds. Dans presque 50% des cas, les tumeurs de notre série prennent l'aspect d'un placard multinodulaire et, en raison du retard de consultation des patients, les lésions sont souvent évoluées avec une taille supérieure à 10 cm chez le 1/3 des patients. L'extension ganglionnaire ou à distance n'a jamais été retrouvée chez les patients de notre étude. Les données cliniques sont présentées dans le Tableau 3 et le Tableau 4.

**Tableau 1:** Délais diagnostiques

Délais diagnostiques	Nombre de cas
0– 1 an	4 cas
1– 5 ans	13 cas
>5 ans	7 cas
Non précisé	8 cas
Total	32 cas

**Tableau 2:** Localisations tumorales

Localisations	Nombre de cas
Scalp	1 cas
Face (prétragiènne)	1 cas
Thorax antérieur	6 cas
Dos	6 cas
abdomen	8 cas
membre supérieur (région scapulaire comprise)	7 cas
membre inférieur	3 cas
Total	32 cas

**Tableau 3:** Aspects cliniques des tumeurs

Aspects cliniques	Nombre de cas
Nodule isolé	8 cas
Placard multinodulaire	14 cas
Lésion ulcéro – bourgeonnante	3 cas
Plaque fibreuse	7 cas
total	32 cas

**Tableau 4:** Taille apparente des tumeurs

Taille apparente de la tumeur	Nombre de cas
0 – 3 cm	5 cas
3 – 10 cm	16 cas
> 10 cm	11 cas
Total	32 cas

**Figure 2:**
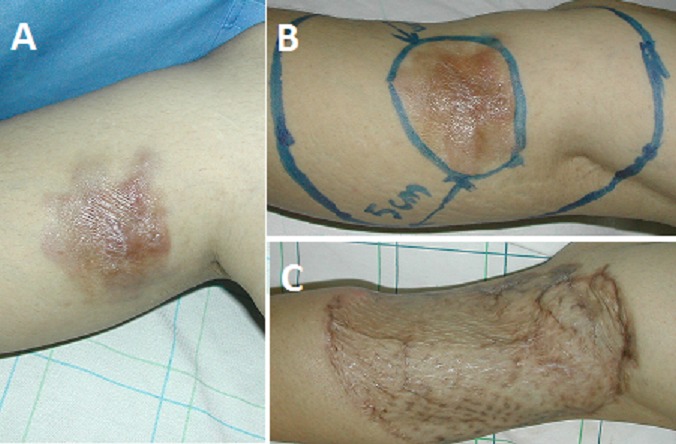
A) DFS de la région postérieure de la jambe droite; B) tracé des incisions pour exérèse de la tumeur avec des marges latérales de 5 cm; C) aspect à J30 post opératoire après exérèse de la tumeur et couverture par greffe cutanée

**Figure 3:**
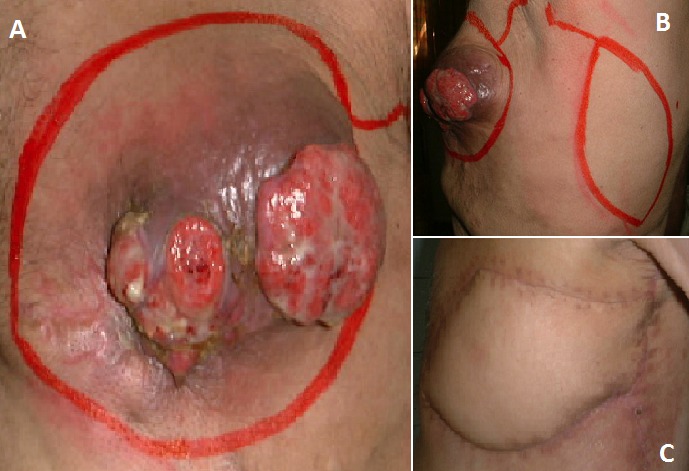
A) DFS d'aspect bourgeonnant de la paroi thoracique latérale; B) tracé des limites de la tumeur et d'un lambeau musculo – cutané du grand dorsal prévu pour la couverture après exérèse; C) aspect à 2 mois post opératoire

Le traitement a été exclusivement chirurgical dans la totalité des cas. Chez tous les patients de notre série, l'exérèse tumorale est passée en surface et latéralement à 5 cm de la lésion visible et palpée. En profondeur, l'exérèse a emporté une barrière anatomique saine (périoste dans la localisation au niveau du scalp, aponévrose musculaire dans les autres localisations). La couverture de la perte de substance résultante de l'exérèse tumorale a été réalisée, dans la quasi-totalité des cas, en différé, après étude anatomopathologique et confirmation histologique du caractère carcinologique de l'exérèse. Elle a fait appel dans la grande majorité des cas à des greffes cutanées facilitant la surveillance et la détection précoce d’éventuelles récidives locales (Figure 2), parfois après réduction de la perte de substance par des points rapprochants. Dans certains cas, et devant le caractère transfixiant de l'exérèse au niveau des parois abdominale et thoracique, la reconstruction s'est faite dans l'immédiat par des lambeaux locorégionaux: lambeau de fascia lata, lambeau pédiculé musculo – cutané du grand dorsal (Figure 3), lambeau musculo-cutané du grand droit de l'abdomen. Une mise en place de plaque prothétique a été réalisée chez deux malades.

Un cas de DFS du scalp a bénéficié d'abord d'une couverture par des lambeaux de transposition du scalp avec greffe cutanée de la zone donneuse, puis devant la récidive 2 ans plus tard, et devant l'insuffisance du scalp restant, l'indication d'un lambeau libre est devenue incontournable. Il a bénéficié d'une couverture par un lambeau musculo – cutané libre du grand dorsal (Tableau 5).

**Tableau 5:** Les différents moyens de couverture utilizes

Moyens de couverture	Nombre de cas
Greffe de peau semi épaisse	21 cas
**Lambeaux loco – régionaux**:	
Tenseur du facia lata	2 cas (DFS paroi abdominale sous
Musculo –cutané pédiculé du grand	ombilicale)
dorsal	2 cas (DFS thorax antérieur)
Fascio – cutané abdominal transverse	1 cas (DFS thorax antérieur)
Lambeaux de transposition du scalp	1 cas (DFS scalp)
Lambeau du grand droit de l'abdomen	1 cas (DFS thorax antérieur)
**Lambeau libre du grand dorsal**	1 cas (après reprise du DFS du scalp)
**Plaque prothétique associée**	3 cas (DFS paroi abdominale sous ombilicale et thorax)

Le suivi des malades, reposant sur l'examen clinique local, général et des aires ganglionnaires avec réalisation de radiographie de thorax, s'est fait de façon régulière avec contrôle trimestriel pendant la première année, puis un contrôle semestriel pendant 2 ans et un contrôle annuel à vie. Le recul moyen de suivi des patients de notre série est de 2,6 ans avec des extrêmes de 1 à 4 ans et sur les 32 cas colligés, 9 ont été perdus de vue pour des raisons probablement socio - économiques ou culturelles. Parmi les 22 patients vus en première intention, 3 ont récidivé (au niveau du scalp, région prétragiènne, région lombaire). Parmi les 10 patients vus en récidive, 5 ont récidivé (au niveau de la région de l’épaule, du dos, de la paroi abdominale sous ombilicale) Le délai moyen de récurrence est de 1 à 3 ans Aucune dégénérescence sarcomateuse, aucune métastase ganglionnaire ou à distance et aucun décès, n'ont été relevés chez les patients de notre série.

Les résultats ont été jugés satisfaisants sur le plan esthétique et fonctionnel. Des complications mineures ont toutefois été notées; il s'agit, chez trois patients, de nécroses partielles de lambeaux qui ont été traitées par cicatrisation dirigée. Chez une patiente, une rétraction axillaire après greffe cutanée d'une perte de substance scapulaire est survenue, elle a été libérée par plastie locale en trident.

## Discussion

De toutes les définitions, celle de DEGOS [3] nous semble la plus complète: ”c'est une tumeur conjonctive dermique à cellules fusiformes, plus ou moins proche par sa structure histologique des tumeurs sarcomateuses, mais qui s'oppose aux fibrosarcomes vrais primitifs par son origine toujours cutanée, et par son évolution très lente. Elle ne comporte qu'exceptionnellement, et à un stade très tardif, une transformation sarcomateuse franchement maligne métastasiante”.

Pour certains auteurs, cette tumeur est plus fréquente chez les africains et la race noire en générale [2, 4]. A propos de l’âge de début, il se situe entre 20 et 50 ans avec des moyennes oscillant entre 28 et 47 ans selon les auteurs, ce qui est en accord avec nos résultats [4]. En ce qui concerne le sexe, certains auteurs ont, en effet, comme nous, retrouvé une prédominance masculine [4, 5], tandis que d'autres [6] ont noté le contraire.

Dans notre série, nous n'avons pas retrouvé de facteur déclenchant (traumatisme, cicatrice…) comme rapporté par certains auteurs [4, 7]. D'autres auteurs évoquent différents facteurs exogènes dans la survenue de l'affection tels que des cicatrices de brûlure [8], de vaccination [8], de radiothérapie [8], des nævi traumatisés [9], des lésions syphilitiques [9], des microtraumatismes sur peau saine [8, 9], des lésions de kératose arsenicale iatrogènes ou professionnelles [4]. Cliniquement, nos résultats rejoignent ceux de la littérature en ce qui concerne la localisation de la tumeur, la taille, l'aspect clinique et le retard à la demande thérapeutique [4–6, 8].

En effet, en ce qui concerne l'aspect clinique: au départ, la lésion se présente comme une plaque indurée, recouverte d'une peau d'aspect et de coloration normale, parfois blanchâtre, blanc-jaunâtre, rosée, violacée ou rougeâtre, elle est apparemment bien délimitée et est mobile par rapport aux plans profonds. A un stade plus ancien, la plaque s’étale, sa surface devient irrégulière et bosselée, réalisant au bout de quelques mois à quelques années, une masse multinodulaire, souvent polychrome, de taille variable, dure, parfaitement mobile sur les plans profonds. Cette évolution en deux stades n'est pas constante car certaines formes sont d'emblée uninodulaires ou multinodulaire avec fusion secondaire des nodules. Des cas de ”tumeurs monstrueuses ” atteignant 6,5 voire 7 kg [8] ont été décrits. Les tumeurs peuvent atteindre des dimensions énormes allant jusqu’à 25 cm de diamètre [4]. La lésion peut se développer en n'importe quelle partie du corps [4]. Dans notre étude, les localisations tumorales intéressent préférentiellement le tronc et les extrémités proximales des membres. Les métastases ganglionnaires et les métastases viscérales sont exceptionnelles et ne peuvent être retrouvées qu'après de longues évolutions et une transformation sarcomateuse [10]. Elles n'ont été retrouvées chez aucun patient de notre série.

Sur le plan anatomo – pathologique, La tumeur se présente sous forme d'une prolifération cellulaire dense, mal limitée, non encapsulée, occupant le derme, le plus souvent dans sa totalité. Elle envoie des prolongements dans l'hypoderme, sans détruire les éléments de celui-ci, tandis que l’épiderme est respecté. Les cellules sont allongées, fusiformes, à cytoplasme plus ou moins abondant, à noyau ovalaire, régulier. Les mitoses sont variables avec de rares atypies. Le stroma est variable d'une zone à l'autre. Les fibres collagènes et réticuliniques sont plus ou moins abondantes, tandis que les fibres élastiques sont refoulées à la périphérie de la tumeur. Au sein des amas de cellules néoplasiques, on distingue un nombre variable d'espaces vasculaires et des coulées cellulaires péri-nerveuses. Dans le temps survient une diminution progressive de la composante fibreuse conjonctive et une augmentation de la densité cellulaire. Sur le plan architectural, les cellules sont disposées en faisceaux rayonnants (aspect en ”rayon de roue”) ou tourbillonnants. Tous nos patients ont bénéficié d'un examen histologique préalable, la lecture est parfois difficile, surtout dans les cas vu en récidive. Le recours à l'immuno – histo – chimie est, dans ces cas difficiles, souvent réalisé.

En ce qui concerne la prise en charge chirurgicale, nous avons opté, comme la majorité des auteurs [4–6, 8, 11], pour une marge de sécurité de 5 cm en superficie avec sacrifice d'une barrière anatomique saine en profondeur. La couverture des pertes de substance engendrées par l'exérèse fait appel aux différents moyens offerts par la chirurgie plastique reconstructrice allant de la greffe cutanée au techniques complexe de microchirurgie par transfert de lambeaux musculo – cutanés libres micro – anastomosés. Cette couverture se fait souvent, comme dans les cas de notre série, en différé après confirmation du caractère carcinologique de l'exérèse par l’étude anatomo – pathologique de la pièce opératoire. Certains auteurs de publications plus récentes [12, 13], préconisent une réduction systématique des marges latérales de recoupes à 3 cm, mais les résultats de ces études ne sont que préliminaires avec un recul minimum faible et restent à évaluer. Plusieurs équipes utilisent la technique de Mohs avec un examen extemporané. Elle consiste à enlever dans un premier temps la majeur partie de la masse tumorale, puis à effectuer des recoupes en congélation sur la face inférieure de la pièce opératoire, ce qui permet de recueillir des lamelles tissulaires horizontales. Après la lecture, les zones envahies sont à nouveau traitées de la même façon jusqu’ à ce que l′on n′observe plus de tissu tumoral sur les coupes.

Les équipes [14–16] utilisant cette technique ont montré que des marges d'exérèse latérales de 3 cm voire 2,5 cm sont suffisantes. Le taux global de récidive pour ces équipes est faible (de l'ordre de 3%) [9–11] Cette technique de Mohs apparait comme très séduisante, mais contraignante, rendant difficile son application en technique de routine Selon REVOL [13], Dans l’état actuel des connaissances, la marge d'exérèse de 5 cm doit s'appliquer par sécurité sur l'ensemble des localisations, sauf les régions péri – orificielles, les seins et les extrémités où les conditions anatomiques imposent une réduction des marges à 3 cm voire même moins. Le contrôle histologique des berges par un anatomopathologiste compétent est alors fondamental. Dans ces localisations, la technique de Mohs mérite d’être discutée, mais dans notre contexte, les conditions actuelles ne permettent pas de l'envisager raisonnablement en raison surtout de la non disponibilité d'anatomo – pathologiste dédié ce genre de technique. En raison de sa faible activité mitotique, le DFS n'est pas radiosensible [4, 8, 10]. Dans notre série, aucun de nos malades n'a bénéficié de RTH. La chimiothérapie n'est pas une méthode efficace, toutefois, un certain espoir avec l'Imatinib (Glivec*) existe, et actuellement, plusieurs études cliniques sur le rôle néo adjuvent de cette molécule sont en cours [17].

Concernant l’évolution, plusieurs publications rapportent la faiblesse des récidives après une chirurgie large d'emblée par rapport aux cas vus en seconde intention [3–5, 8]. Ceci se confirme dans notre série, puisque les récidives n'ont en effet concerné que 13,6% des patients vus en 1^ère^ intention alors la récidive est survenue chez 50% des patients vus en récidive.

## Conclusion

Sur le plan épidémiologique et clinique les données de notre série confirment celles de la littérature. Sur le plan évolutif: Cette tumeur engage rarement le pronostic vital par sa seule prolifération, par contre, le pronostic est lié à son caractère destructeur localement et au risque de récidive qui reste principalement en rapport avec la qualité de la première exérèse. Les possibilités de guérison en cas de chirurgie primaire bien conduite sont significativement supérieures à celles d'une chirurgie de rattrapage. L'exérèse tumorale doit donc être large en surface, passant à 5cm de la lésion et profonde avec sacrifice d'une barrière saine en profondeur. La réduction systématique des marges cliniques d'exérèse en surface à moins de 5 cm fait courir inutilement un risque de récidive au patient et doit être réservée à certaines localisations anatomiques difficiles. L'amélioration du pronostic des DFS nécessite: un diagnostic précoce afin d'assurer une prise en charge rapide des patients et donc d’éviter des exérèses mutilantes dont la réparation est parfois complexe. D'où l'intérêt de la sensibilisation et de la formation des médecins généralistes pour l'orientation rapide des malades vers des centres spécialisés; une prise en charge multidisciplinaire, impliquant différents spécialistes (dermatologues, chirurgiens plasticiens et anatomopathologistes compétents).
